# Prostate Cancer Patients' Refusal of Cancer-Directed Surgery: A Statewide Analysis

**DOI:** 10.1155/2015/829439

**Published:** 2015-04-20

**Authors:** K. M. Islam, Jiajun Wen

**Affiliations:** Department of Epidemiology, College of Public Health, University of Nebraska Medical Center, Omaha, NE 68198, USA

## Abstract

*Introduction*. Prostate cancer is the most common cancer among men in USA. The surgical outcomes of prostate cancer remain inconsistent. Barriers such as socioeconomic factors may play a role in patients' decision of refusing recommended cancer-directed surgery. *Methods*. The Nebraska Cancer Registry data was used to calculate the proportion of prostate cancer patients recommended the cancer-directed surgery and the surgery refusal rate. Multivariate logistic regression was applied to analyze the socioeconomic indicators that were related to the refusal of surgery. *Results*. From 1995 to 2012, 14,876 prostate cancer patients were recommended to undergo the cancer-directed surgery in Nebraska, and 576 of them refused the surgery. The overall refusal rate of surgery was 3.9% over the 18 years. Patients with early-stage prostate cancer were more likely to refuse the surgery. Patients who were Black, single, or covered by Medicaid/Medicare had increased odds of refusing the surgery. *Conclusion*. Socioeconomic factors were related to the refusal of recommended surgical treatment for prostate cancer. Such barriers should be addressed to improve the utilization of surgical treatment and patients' well-being.

## 1. Introduction

Prostate cancer is the most common cancer among men in the United States, with estimated incident cases of 209,292 men being diagnosed in 2011, and 27,970 died from prostate cancer in that year [1], accounting for 14% of all new cancer cases and 5% of all cancer deaths [2]. It is also the most prevalent cancer among men in the state of Nebraska. During the period between 1995 and 2012, the Nebraska Cancer Registry recorded 22,335 prostate cancer incident cases. Although prostate cancer is most frequently found among men over 50 years old, it is often caught at early stages by screening, and these early-stage cases are expected to live relatively long if treated correctly. This makes patients with localized and regional prostate cancer have a five-year survival rate of almost 100%, but it drops dramatically to 26% among patients with distant stage prostate cancer [2].

Recommended treatment options for prostate cancer include radical prostatectomy (RP) and external-beam radiation therapy (EBRT) for patients with early stages and hormonal manipulations, bisphosphonates, EBRT with/without hormonal therapy and palliative surgery, or radiation therapy for late-stage patients [3]. However, although the treatment guideline for prostate cancer is well defined, the outcomes of the treatment still remain controversial. In a literature review of localized cases, in which the 10-year cancer-specific survival was compared for radical prostatectomy, radiation therapy, and deferred treatment, it turned out that the surgical approach had the best survival rate (about 93%) [4]. However, the Prostate Intervention Versus Observation Trial (PIVOT), which directly compared radical prostatectomy with watchful waiting, suggested that the 10-year survival rates were not significantly different for prostatectomy and watchful waiting (hazard ratio [HR] is 0.88; 95% CI, 0.71–1.08; *p* = 0.22), and there was no significant difference in prostate cancer-specific mortality for these two approaches as well (HR, 0.63; 95% CI, 0.36–1.09; *p* = 0.09) [5]. For late-stage patients, study showed surgery can only serve as a palliate care, and no significant benefit for overall survival was observed after the surgery [6].

The risk of impaired urinary and sexual function also leads to conservation on surgical treatment for clinically localized prostate cancer. The Prostate Outcome Study pointed out that, at 18 or more months following radical prostatectomy on localized prostate cancer patients, 8.4% of men were incontinent and 59.9% were impotent, suggesting the surgery was associated with significant erectile dysfunction and some decline in urinary function [7]. This effect was observed in the long term that a small portion of patients experienced changes in urinary or sexual function including frequent urinary leakage and infirm erections between years 2 and 5 after prostatectomy, while functional outcomes remained relatively stable in the majority of patients [8].

The expense of prostate cancer treatment, especially the surgical approaches, may also be a concern. A study estimating the health care cost for prostate cancer treatment based on SEER-Medicare data suggests that prostate cancer accounted for the most of Medicare expenditures for male in 1996 and was the fourth most expensive single cancer according to the national health expenditure, which could be attributed to high incidence of prostate cancer due to the maturity of screening [9]. It was estimated that the average direct medical cost of prostate surgery, including three types of radical prostatectomy (open, laparoscopic-assisted, and robotic-assisted), ranges from $6,042 to $10,684, and the mean lifetime cost for surgery ranges from about $20,000 to $36,000 for clinical localized prostate cancer patients [10]. Although this study also showed the cancer-directed surgery results in on average more than ten-year survival after the surgery among localized patients, it also brings patients complications such as ejection dysfunction and urinary incontinence, leading to a reduction in quality of life. Also, it is reported that surgery-associated complications such as 30-day postoperative mortality, major acute surgical complications, longer hospital stays, and higher rates of rehospitalization were observed [11, 12]. Both the expenses for the surgical treatment and the complications that the surgery may bring can prevent early-stage patients from seeking cancer-directed surgery.

In this study, we were interested in (1) the year trend of the proportion of patients who were recommended the prostate cancer-directed surgery and the refusal rate of the surgery and (2) socioeconomic factors that were related to the refusal of surgery.

## 2. Materials and Methods

### 2.1. Data Source

The data used in the analyses was a subset of the Nebraska Cancer Registry (NCR) data, including all prostate cancer incidences recorded by the cancer registry from year 1995 to the end of 2012.

The cancer registry records the reason for no cancer-directed surgery based on the COC standard as a routine. We recoded the reasons into two categories: cancer-directed surgery was recommended but refused by the patient (or the family member/guardian) or surgery was recommended and administered/not administered due to other reasons, with the latter one including the situation that the patient died prior to the planned surgery; it was unknown if the recommended surgery was actually administered or the recommended surgery was not administered for unknown reasons. This simplified dichotomy briefly summarized the possible situations that the patient would encounter while making a decision for the recommended surgery. That is, in general, all patients included in our study were recommended the cancer-directed surgery, and then they were only distinguished by whether they willingly refused the treatment.

**Figure 1:**
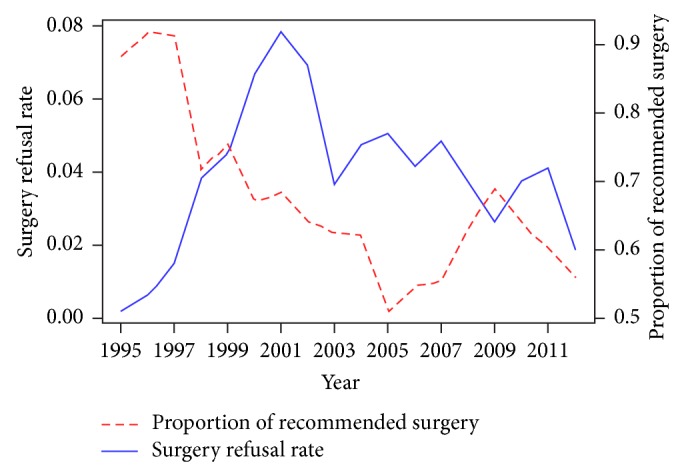
The annual proportion of prostate cancer patients recommended the cancer-directed surgery and the annual surgery refusal rate.

**Figure 2:**
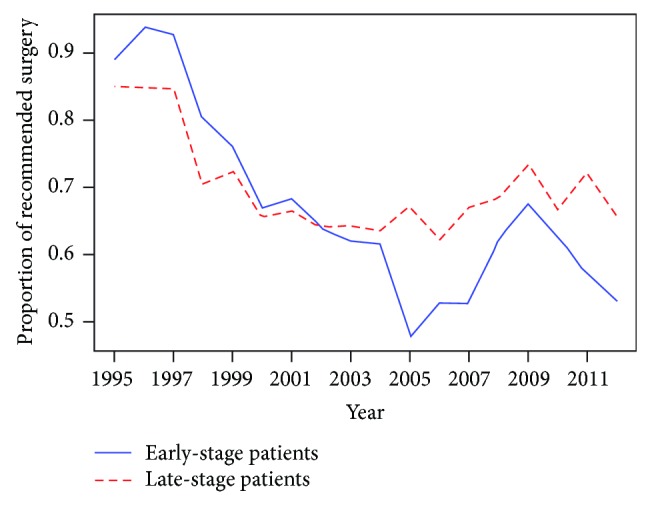
The annual proportion of early-stage/late-stage prostate cancer patients recommended the cancer-directed surgery.

We calculated the following descriptive statistics over year: the annual proportions of prostate cancer patients recommended the cancer-directed surgery (the number of patients recommended surgery in that year/total number of prostate cancer patients in that year), the annual surgery refusal rates (the number of patients refused recommended surgery in that year/total number of patients recommended the surgery in that year), and the annual proportions of early-stage/late-stage patients recommended the surgery (the number of early-stage or late-stage patients recommended surgery in that year/total number of early-stage or late-stage prostate cancer patients in that year) and we illustrated the statistics in Figures 1 and 2.

### 2.2. Socioeconomic Indicators

The NCR database also recorded demographic and socioeconomic indicators of cancer patients as a routine. In our study, we investigated what demographic and socioeconomic variables were significantly related with the refusal of cancer-directed surgery among Nebraska patients. The variables included patients' age, race (White/Black/other), ethnicity (Hispanic or Latino/non-Hispanic or non-Latino), marital status at diagnosis (single/married), primary payment methods (no insurance/Private insurance/Medicaid/Medicare/other), and residential status (urban/rural/unknown), all controlling for prostate cancer stage at diagnosis (in situ or localized/regional/distant). The adjusted odds ratio for each variable was calculated with Wald 95% confidence interval controlling for other variables simultaneously.

## 3. Results

### 3.1. Year Trend for Recommendation and Refusal of Prostate Cancer-Directed Surgery

We identified 22,335 patients with prostate cancer as their primary diagnosis between year 1995 and the end of 2012 according to the NCR database. Among these patients, 14,876 patients were recommended the prostate cancer-directed surgery, and we took this subset as our sample of interest.

Figure 1 shows that, from year 1995 to the end of 2012, the refusal rate of cancer-directed surgery reached its peak in 2001 and then had been fluctuating, with a slight decrement in trend over recent years. The overall refusal rate of surgery was 3.9% over the 18 years. The refusal rate accreted and reached 7.3% (69 out of 945) in 2001 and then decreased sharply over the following two years and remained fluctuating from 2003 to 2012 with a slight trend of decreasing.

Figure 2 shows that the trends of the annual proportions of patients recommended the cancer-directed surgery for prostate cancer were very similar between early-stage patients and late-stage patients over the 18 years, indicating there was an overall effect that influenced both early-stage patients and late-stage patients with regard to the recommendation of the surgery as a treatment approach. The proportions of patients recommended the surgery were as high as 90% before 1996 and went remarkably downward after then and reached the nadir in years 2005 and 2006. Over recent years, the annual proportions of recommended surgery fluctuated around 60 percent for early-stage patients and 70 percent for late-stage patients. It was also illustrated that the reduction of the proportion of recommended surgery within late-stage patients was smoother than that within early-stage patients.

### 3.2. Characteristics of Patients Undergoing/Refusing Cancer-Directed Surgery

Among 14,876 prostate patients in Nebraska recommended cancer-directed surgery as their treatment, five hundred and seventy-nine (579) patients refused the surgery. The distributions of stage at diagnosis, demographic, and socioeconomic variables were summarized in Table 1. Under univariate analyses, chi-square test was used to investigate the association between each demographic and socioeconomic variable and whether patients refused the recommended cancer-directed surgery. The average age of patients who refused the cancer-directed surgery was 69.66 with standard deviation equal to 8.48 years, and average age of those who underwent the surgery was 66.93 years with standard deviation equal to 9.81 years. Two-sample *t*-test with unequal variances indicated patients who refused recommended surgery were significantly older than those who underwent the surgery (*p* < 0.0001). The proportions of patients refusing the surgery were significantly different across the stages at diagnosis (*p* < 0.0001). Patients diagnosed at early stages (in situ/localized) turned out to have the highest proportion (4.58%) of refusing the surgery, followed by that of patients diagnosed at a distant stage (3.12%), and the proportion of refusal among patients diagnosed with a regional stage appeared to be the lowest (0.66%). The univariate analyses also suggested there were significant effects of patients' race (*p* = 0.024), primary payment methods (*p* < 0.0001), and marital status (*p* = 0.0003) on the decision of refusing cancer-directed surgery, while patients' ethnicity (*p* = 0.424) and the residential status (*p* = 0.058) turned out not to be related to the decision of refusing surgery.

### 3.3. Adjusted Odds Ratios of Refusing Recommended Surgery

Further investigation using multivariate logistic regression estimated the adjusted odds ratios with 95% Wald confidence intervals for these demographic or socioeconomic variables. The modeling was conducted by a two-step approach. At the first step, all variables including stage at diagnosis were included in the initial model, and patients' ethnicity (*p* = 0.079) and residential status (*p* = 0.145) were excluded from the model because of insignificant effects. Patients' age also appeared to be insignificant (*p* = 0.234) but was remained so that the effect of age could be controlled while studying other variables. At the second step, the general logistic model was rerun with the remaining variables and all significant effects were kept as our final model (Table 2). In this case, the adjusted odds of refusing cancer-directed surgery among Black patients were 2.05 times those of White patients (*p* = 0.001; 95% CI: 1.334–3.158), while there were no significant differences in the odds of refusal between White patients and patients of other races (*p* = 0.364). Early-stage patients were significantly more likely to refuse the recommended surgery compared to patients with regional stage (*p* < 0.0001; OR = 6.993; 95% CI: 4.237–11.494), yet patients with early stages and with distant stage did not differ in the proportion of surgery refusal (*p* = 0.07). Governmental insurances, that is, Medicaid and Medicare, were found to be significantly related to higher odds of surgery refusal compared to Private insurance as primary payment methods, with adjusted OR that equaled 3.497 and 2.319, respectively. Being single at the time of diagnosis (never married/widowed/divorced/separate) was related independently to an increment of surgery refusal after adjusting for other factors (OR = 1.361; *p* = 0.004; 95% CI: 1.106–1.672).

### 3.4. Adjusted Odds Ratios for Early-Stage Patients

Because, in the univariate analyses, the proportion of refusing cancer-directed surgery among prostate cancer patients diagnosed at early stages (in situ/localized) turned out to be significantly higher compared to patients diagnosed with later stages (regional/distant), we further investigated the effects of demographic and socioeconomic variables on the decision-making among early-stage prostate cancer patients. The same approach was used as the previous analysis and the effects of patients' race (*p* = 0.003), primary payment methods (*p* < 0.0001), and marital status (*p* = 0.01) still turned out to be significant if the patients were diagnosed at early stages (Table 3).

## 4. Discussion

The screening and treatment of prostate cancer have developed maturely over the last 30 years. In the 90s, early detection approaches such as prostate-specific antigen (PSA) blood were acknowledged to be effective in finding early-stage prostate cancer and are recommended among population at risk [13, 14], and the incidence rate of prostate cancer diagnosed in USA reached its peak [15]. However, in 2012, the U.S. Preventive Services Task Force released statement recommending against PSA-based prostate cancer screening based on the evidence that a substantial percentage of men who had asymptomatic cancer detected by PSA screening had a tumor that either would not progress or would progress slowly and remain asymptomatic for the man's lifetime [16]. In the meantime, the incidence rate has remained consistent until recent years, and with the advance in cancer treatment, most prostate cancers, especially in their early stages, can be cured by cancer-directed surgery, and therefore the quality of life and survival are improved by the surgery.

The decrease of recommended surgical treatment for both early- and late-stage prostate cancer initiated during the late 90's. This decrease reflects physicians' decision after valuing the advantages and disadvantages of the surgical treatment such as the limited survival benefit and functional sequelae. Besides, the emergence of new noninvasive detection technology such as magnetic resonance imaging (MRI) and proton magnetic resonance spectroscopy (1H MRSI) has become the most sensitive evaluation for prostate cancer and could help physicians monitor the progress of patients who select watchful waiting or minimally aggressive cancer therapies to avoid surgical treatment [17]. Along with the decrease in surgery recommendation, patients' decision of refusing cancer-directed surgery increased. Based on this study, the overall refusal rate of surgery was 3.9% over the 18 years, and it reached 7.3% in 2001. Among cancers that can easily be detected and treated by cancer-directed surgery at early stages, the surgery refusal rate for prostate cancer is exorbitant. A recent study pointed out that a chart review in Northern Alberta Health Region (NAHR) showed that during a 26-year period breast cancer had only 1.2% of refusal rate for standard treatment including surgery [18]. The Nebraska Cancer Registry data also showed that, in the same time period as in our study (1995–2012), prostate cancer had the highest refusal rate of recommended surgery among the top five prevalent cancers within the state, compared to lung cancer (3.3%), breast cancer (0.36%), colorectal cancer (0.55%), and skin melanoma (0.08%).

We conjecture that the trend in refusal of recommended surgical treatment may be attributable to (1) the improved detection methods for both early- and late-stage prostate cancer, (2) limited benefit that could be obtained from cancer-directed surgery such as survival within early-stage patients, (3) risk of functional sequelae of the surgery such as impaired urinary and sexual function, and (4) high cost of cancer-directed surgery.

The strongest predictor of declining prostate cancer-directed surgery was cancer stage at diagnosis. Patients diagnosed as localized and distant were more likely to refuse the recommended surgery, compared to patients diagnosed as regional. It is understandable for early-stage patients to refuse the cancer-directed surgery, given that the surgery may not improve survival rate but may bring surgery-related complications [11, 12]. For patients diagnosed with distant stage, given the patients are on average older and have impaired 5-year survival, the reason for declining surgery may be due to avoiding suffering from surgery-associated complications and certain socioeconomic determinants. However, in our study, due to small sample size for patients with a distant stage declining surgery, we were unable to further mine the related social factors for these patients.

A close look at the demographic and socioeconomic status distribution of patients who declined the recommended surgery, especially among early-stage patients, suggested that patients being single or minority, with governmental insurance as primary payment method, were at higher chance of declining the cancer-directed surgery, and same phenomenon can be observed among patients with early-stage prostate cancer. This suggested that racial and socioeconomic differences may play a role in the decision of declining the recommended surgery for prostate cancer patients. Race was widely acknowledged in previous studies as a strong predictor of initial prostate cancer treatment [19–24], and minorities such as Black patients were less likely to undergo surgery but more likely to receive conservative treatment. Our study confirmed the finding that Black patients had approximately twice the odds of refusing the cancer-directed surgery compared to White patients, while the information from other racial groups was insufficient and therefore was not tested. Marital status had been studied before to be related to cancer stage at diagnosis and treatment in cancers of other types [25, 26], but little research had been done concerning prostate cancer-directed surgery. Our study extended the findings by showing that being married was related to lower odds of refusing surgical treatment, yet the outcomes following the surgery remained unknown and should be further addressed. Our study also found that patients with Medicaid/Medicare coverage were more likely to refuse the cancer-directed surgery, and this could be explained in two ways. One explanation is that patients may become eligible for Medicaid as a result of poor health [27] and were more likely to be diagnosed at clinical-advanced stage. In our case, this theory may help explain the refusal of surgery in late-stage patients but cannot explain the fact that early-stage patients had the highest rate of refusing recommended surgery. Rowland and Lyons [28] also argued that although Medicare coverage offered basic health insurance to promote access to care yet for those who had the most health needs, in this case patients who needed care for cancer, financial concerns could impede access to needed medical care such as cancer-directed surgery. Another explanation could be the disparity in the quality of treatment and care received through different insurances, and, for patients with the same other characteristics, more aggrieve treatments were more likely to be given to those with Private insurance compared to those with Medicare or Medicaid as their primary payment method [26].

A limitation of this study is that it failed to take some other important socioeconomic factors into consideration due to the incomplete information that could be drawn from the database. Socioeconomic indicators such as education and income level were not recorded by the cancer registry, while the population-level approximation of income could be obtained if linked to census tract poverty level. Some researchers believe that different choice of socioeconomic indicators may lead to disparate conclusions, and potential confounding factors should be considered; therefore, the selection of the underlying social determinants, especially those indicating income equality, should be scrupulously conducted [29].

## 5. Conclusions

The recommendation rate of prostate cancer-directed surgery had been decreasing for both early- and late-stage patients from 1995 to 2012 in Nebraska. The refusal rate of recommended surgery reached the top of 7.9% in 2001 and then had been decreasing over the following 11 years. Patients diagnosed with in situ and localized tumor were most likely to refuse the recommended surgery, followed by patients diagnosed with distant tumor. Black and single patients, as well as patients with governmental insurance as the primary payment method, were more likely to refuse the recommended surgery. The nonclinical barriers should be addressed to reduce the nonclinical factors to reduce the disparities in prostate cancer treatment and outcomes.

## Figures and Tables

**Table 1:** Characteristics of patients undergoing/refusing the prostate cancer-directed surgery 1995–2012.

	Underwent surgery (*n* = 14297)	Refused surgery (*n* = 579)	Total (*n* = 14876)
^*^Age	66.93 ± 9.81	69.66 ± 8.48	67.04 ± 9.78
^*^Stage at diagnosis			
In situ/localized	11,446 (80.06)	549 (94.82)	11,995 (80.63)
Regional	2,416 (16.90)	16 (2.76)	2,432 (16.35)
Distant	435 (2.92)	14 (2.42)	449 (3.02)
^*^Race			
White	13,633 (95.36)	539 (93.09)	14,172 (95.27)
Black	367 (2.57)	25 (4.32)	392 (2.64)
Other	297 (2.08)	15 (2.59)	312 (2.10)
Ethnicity			
Hispanic/Latino	428 (2.99)	14 (2.42)	442 (2.97)
Non-Hispanic/Non-Latino	13,869 (97.01)	565 (97.58)	14,434 (97.03)
^*^Insurance type			
No insurance	490 (3.43)	5 (0.86)	495 (3.33)
Private insurance	3,012 (21.07)	75 (12.95)	3,087 (20.75)
Medicaid	57 (0.40)	7 (1.21)	64 (0.43)
Medicare	5,350 (37.42)	346 (59.76)	5,696 (38.29)
Other insurance types	5,388 (37.69)	146 (25.22)	5,534 (37.20)
^*^Marital status at diagnosis			
Single	2,360 (16.51)	132 (22.80)	2,492 (16.75)
Married	11,219 (78.47)	418 (72.19)	11,637 (78.23)
Unknown	718 (5.02)	29 (5.01)	747 (5.02)
Residency			
Urban	5,654 (39.55)	212 (36.61)	5,866 (39.43)
Rural	7,352 (51.42)	299 (51.64)	7,651 (51.43)
Unknown	1,291 (9.03)	68 (11.74)	1,359 (9.14)

^*^Indicates an unadjusted statistically significant effect (*p* < 0.05) exists within the variable.

**Table 2:** Adjusted odds ratio estimates and Wald confidence intervals for patients of early and late stages.

Effect	Odds ratio estimates	95% confidence limits		*p* value
Age	1.006	0.996	1.017	0.234
Black versus White	2.052	1.334	3.158	0.001
Other races versus White	1.286	0.747	2.212	0.364
Regional versus in situ/localized	0.143	0.087	0.236	<0.0001
Distant versus in situ/localized	0.604	0.350	1.043	0.07
No insurance versus Private insurance	0.376	0.151	0.940	0.036
Medicaid versus Private insurance	3.497	1.506	8.121	0.004
Medicare versus Private insurance	2.319	1.725	3.118	<0.0001
Other methods versus Private insurance	0.958	0.711	1.292	0.779
Married versus single	0.735	0.598	0.904	0.004
Unknown versus single	0.856	0.557	1.316	0.479

**Table 3:** Adjusted odds ratio estimates and Wald confidence intervals for early-stage patients.

Effect	Odds ratio estimates	95% confidence limits		*p* value
Age	1.007	0.996	1.018	0.205
Black versus White	2.103	1.352	3.273	0.001
Other races versus White	1.347	0.781	2.323	0.284
No insurance versus Private insurance	0.390	0.156	0.977	0.045
Medicaid versus Private insurance	3.801	1.619	8.921	0.002
Medicare versus Private insurance	2.248	1.661	3.043	<0.0001
Other methods versus Private insurance	0.946	0.697	1.283	0.719
Married versus single	0.721	0.584	0.892	0.003
Unknown versus single	0.799	0.512	1.246	0.323
